# Thin Films Deposition of Ta_2_O_5_ and ZnO by E-Gun Technology on Co-Cr Alloy Manufactured by Direct Metal Laser Sintering

**DOI:** 10.3390/ma14133666

**Published:** 2021-06-30

**Authors:** Diana-Irinel Băilă, Cătălin Vițelaru, Roxana Trușcă, Lidia Ruxandra Constantin, Ancuța Păcurar, Constantina Anca Parau, Răzvan Păcurar

**Affiliations:** 1 Department of Manufacturing Engineering, Faculty of Industrial Engineering and Robotics, Polytechnic University of Bucharest, Splaiul Independenţei nr. 313, Sector 6, 060042 Bucharest, Romania; roxana.trusca@upb.ro; 2 National Institute for Research and Development in Optoelectronics, Atomiștilor 409, 077125 Măgurele, Romania; catalin.vitelaru@inoe.ro (C.V.); lidia.constantin@inoe.ro (L.R.C.); anca.parau@inoe.ro (C.A.P.); 3 Department of Manufacturing Engineering, Faculty of Industrial Engineering, Robotics, Management and Production Management, Technical University of Cluj-Napoca, B-dul Muncii 103-105, 400641 Cluj-Napoca, Romania; ancuta.costea@tcm.utcluj.ro

**Keywords:** thin films, direct metal laser sintering, DMLS, Co-Cr alloy, e-gun, surface characterization

## Abstract

In recent years in the dental field, new types of materials and techniques for the manufacturing of dental crowns and analog implants have been developed to improve the quality of these products. The objective of this article was to perform the surface characterization and determine the properties of Co-Cr alloy samples fabricated by the direct metal laser sintering (DMLS) process and coated by e-gun technology with thin films of Ta_2_O_5_ and ZnO. Both oxides are frequently used for dental products, in pharmacology, cosmetics, and medicine, due to their good anticorrosive, antibacterial, and photo-catalytic properties. Following the deposition of thin oxide films on the Co-Cr samples fabricated by DMLS, a very fine roughness in the order of nanometers was obtained. Thin films deposition was realized to improve the hardness and the roughness of the Co-Cr parts fabricated by the DMLS process. Surface characterization was performed using SEM-EDS, AFM, and XRD. AFM was used to determine the roughness of the samples and the nanoindentation curves were determined to establish the hardness values and modulus of elasticity.

## 1. Introduction

In Dentistry 4.0, biomaterials are realized by novel and special methods to resist the biological reactions that occur in medical implants (protein adsorption, cell adhesion, cell growth, blood compatibility, etc.), as considered by Dobrzański [1]. Okazaki determined the chemical, physical, and mechanical properties and microstructures of laser-sintered Co-25Cr-5Mo-5W (SP2) and W-free Co-28Cr-6Mo alloys for dental applications [2]. The most important surface properties of these biomaterials are physical properties, durability, and biocompatibility. The physical properties of a biomaterial that are required to be determined are the mechanical strength, permeability, and elasticity of the material [3]. Biocompatibility is the response of a biomaterial or medical device to proteins, cells, and organisms when implants are in contact with biological tissue. Common methods used to characterize biomaterial surfaces are SEM, EDS, XRD, contact angles, ESCA (XPS), auger electron spectroscopy, SIMS, FTIR-ATR, and STM. The direct metal laser sintering (DMLS) process has been developed for industrial and medical applications due to its remarkable capabilities, such as building complex parts that are otherwise difficult to manufacture by conventional methods [4,5,6,7,8]. DMLS is a layer-by-layer automated fabrication process used for producing three-dimensional physical scaled objects directly from 3D CAD data without utilizing part-dependent implements [9]. Han realized a comparative analysis of mechanical properties and metal-ceramic bond strength of Co-Cr dental alloy fabricated by different manufacturing processes [10].

Direct metal laser sintering technology can be used not only in medicine, but also in aerospace, aeronautics, automotive, and electronic industrial domains. In medicine, the DMLS process is successfully used for manufacturing dental restorations, such as dental crowns, bridges, and chapels [11,12,13,14,15,16]. The Co-Cr alloy powder used in the DMLS process presents good engineering properties as well as good corrosion resistance [17,18]. The Co-Cr alloys and Ti alloys are frequently used for implants manufacturing, as these materials are biocompatible and not toxic to the human body.

All parts manufactured by DMLS technology present porous surfaces with powder grains that are impossible to be removed completely. Because of the porosity, the Co-Cr alloy parts realized by DMLS technology do not allow good cleaning (such as glass) [19]. In this article, surfaces were covered with thin oxide layers using e-gun technology to obtain a uniform surface with fine roughness and improved mechanical properties, which allow good cleaning (such as glass) and sterilization [17,18,19].

ZnO films are used in medicine because these materials possess better anticorrosive, antibacterial, and photocatalytic properties. Hacini analyzed the compositional, structural, morphological, and optical properties of ZnO thin films prepared by the PECVD technique [20,21]. Horandghadim N. studied the effect of Ta_2_O_5_ content on the osseointegration and cytotoxicity behaviors in hydroxyapatite-Ta_2_O_5_ coatings applied by EPD on superelastic NiTi alloys and remarked an improvement in implant osseointegration in tissue [22]. Luang H. established the antibacterial properties and cytocompatibility of tantalum oxide coatings necessary to realize implants and medical devices [23]. Pham V. determined the utility of tantalum (Ta) coating to improve surface hardness, impacting the in vitro bioactivity and biocompatibility of Co-Cr [24]. Ta_2_O_5_ films have rapidly evolved in medicine research with the promise of the development of new types of biomaterials used in medicine. Ta_2_O_5_ coatings have an excellent biocompatibility, good dielectric properties, and high corrosion resistance [25]. E-gun technology permits corrosion resistance and uniform thin coatings of the angstrom order [26]. In general, physical vapor deposition methods are used for the optical coatings in the field of ophthalmic and precision optical coatings [27]. These types of methods are applied for aesthetic on anticorrosion and mechanical films.

In this study, we used a novel method based on e-gun technology to realize thin-film coating with Ta_2_O_5_ and ZnO materials on Co-Cr probes sintered by the DMLS process, which were then subjected to surface characterization. Coatings based on Ta and Zn materials are important for improving the surface hardness and surface roughness, which lead to an improvement in in vitro bioactivity and biocompatibility of Co-Cr implants (used mainly in dentistry). Both the roughness of the realized implants and the hardness of the material are highly important since these characteristics significantly influence the osseointegration process of implants into the bone tissue at the end [28,29]. In Dentistry 4.0, the dental implants fabricated using Co-Cr by DMLS process must present better osseointegration in the bone tissue. Therefore, the main objective of this paper was focused on finding the proper methods for improving the hardness and roughness of Co-Cr implants manufactured by the DMLS process, by realizing thin films coated with Ta_2_O_5_ and ZnO in different percentages by e-gun technology to determine the efficiency of the coating process and the optimum ratio that has a significant influence on these important characteristics. This will influence the further osseointegration process of implants into the bone tissue.

## 2. Materials and Methods

### 2.1. Co-Cr Samples Made by DMLS Process and Coated with Ta_2_O_5_ by E-Gun Technology

Six disk samples of Φ10 × 2 mm were designed using SolidWorks 2018 program made of Dassault Group company (Vélizy-Villacoublay, France). The sintered samples were 3D-printed from Co-Cr powder (ST2724G) by the direct metal laser sintering (DMLS) process. The Co-Cr alloy powder used in this research presents the following chemical composition: Co 54.31%, Cr 23.08%, Mo 11.12%, W 7.85%, Si 3.35%, and Mn, Fe < 0.1, determined by Phenix Systems [30].

The engineering properties of Co-Cr alloys (ST2724G) provided by Phenix Systems are: elastic limit 0.2% Rp0.2 = 815 MPa; elongation at break = 10%; Vickers hardness = 375 HV 5; elastic modulus = 229 GPa; mass volume = 8.336 g/cm^3^; corrosion resistance < 4 μg/cm^2^; thermal expansion coefficient = 14.5 × 10^−6^ K^−1^ [31].

The manufacturing system used for DMLS process was the Phenix Systems dental machine type PHX Pro200 Dental (Riom, France) with a fiber laser (P = 50 W, λ = 1070 nm) and with a manufacturing volume of 100 mm × 100 mm × 80 mm. The machine software used for experiments was Phenix Dental (Riom, France). The sintering temperature was 1300 °C and nitrogen gas was used in the process. The thickness of the deposited powder layer was 20 μm and the scanning speed was 0.06 m/s. Linear energy density (LED), which was defined by the ratio of laser power to the scanning speed, was used to tailor the laser sintering mechanism. The value of LED was between 3400 and 6000 J/m [30].

Three disk samples were post-treated in a furnace for 30 min at 900 °C. For preparing the disk samples for e-gun deposition, they were polished and cleaned with ethylic alcohol. The disk samples were mounted on the dome chamber and were cleaned in plasma glow discharges.

For thin films deposition, we used zinc oxide nanopowder (CAS No. 1314-13-2, Sigma-Aldrich (St. Louis, MO, USA), code 544906-10G) and tantalum (V) oxide 99% nanopowder (CAS No. 1314-61-0, Sigma-Aldrich (St. Louis, MO, USA), code 303518-25G). These powders were transformed in target forms necessary for e-gun technology by compaction. The e-gun process was realized in a physical vaporous deposition chamber in a vacuum, using a pressure of 10^−5^ to 10^−6^ torr. The material was vaporized thermally using two electron guns. The vaporization process was realized in a high vacuum that reduced the disk sample contamination. The source material used for the evaporation process was granulated in a solid form at high purity (99.9%).

The physical vaporous deposition chamber was equipped with two e-beam gun heads, one for Ta_2_O_5_ and the other for ZnO. One quartz crystal microbalance controlled the coating growth to report the thickness and evaporating rate. The ion gun was used to increase the density of coating material. The thermal evaporation determines the transition from a solid to vaporous state, using an electron bombardment at 180 °C. The important parameters of coating technology are the average speed of the evaporated particles and the angular distribution. The coating film performed by e-gun technology permits the metal and dielectrics deposition, with high purity, the average speed being in between 10 and 100 A/s and the temperature being 3000 °C in this case.

### 2.2. SEM and EDS Analyses of Co-Cr Samples Fabricated by the DMLS Process and Coated with Ta_2_O_5_ by E-Gun Technology

SEM and EDS techniques were used to investigate the coated, sintered samples. The morphological investigations and the chemical element analyses of samples were performed using a scanning electron microscope QUANTA INSPECT F type with a field emission gun (FE-SEM) (Hillsboro, OR, USA) and a resolution of 1.2 nm, coupled with an energy-dispersive X-ray spectrometer (Hillsboro, OR, USA) with a resolution of 133 eV at MnK. The areas of interest were analyzed qualitatively by microcompositional X-ray spectrometry.

### 2.3. XRD Analysis

XRD analysis was performed by means of X Rigaku-Miniflex II X-ray diffractometer (Austin, TX, USA) that uses CuKα characteristic radiation and a wavelength of λ = 1.5418 Å. Diffraction pattern acquisition was performed in Bragg-Brentano geometry. The features of the diffractometer were: focusing size of 1.0 × 10.0 mm, scanning mode of 2θ, scanning interval of 10°–100°, scanning speed (2θ) of 0.1°/min, and scanning step (2θ) of 0.01°.

### 2.4. AFM Analysis of the Surface Morphology and Roughness

The tapping scans were performed with a Veeco atomic force microscope (AFM) on an area of 5 and 10 μm^2^, with a scanning speed of 0.3 Hz and a resolution of 512 pixels. An RTESPA needle was used for performing the analyses.

### 2.5. Hardness and Modulus of Elasticity Determination Using Nanoindentation Method

Nanoindentation tests were performed by using a Hysitron TI Premier nanoindenter (Billerica, MA, USA) equipped with a 100 nm radius Berkovich diamond tip. The indents were located at least 7.5 μm apart to avoid possible interference effects between the indentation points. The values of H and Er were extracted from load–displacement curves according to the Oliver-Pharr formalism [32].

## 3. Results and Discussions

### 3.1. Co-Cr Samples Fabricated by the DMLS Process and Coated with Ta_2_O_5_ by E-Gun Technology

**Figure 1:**
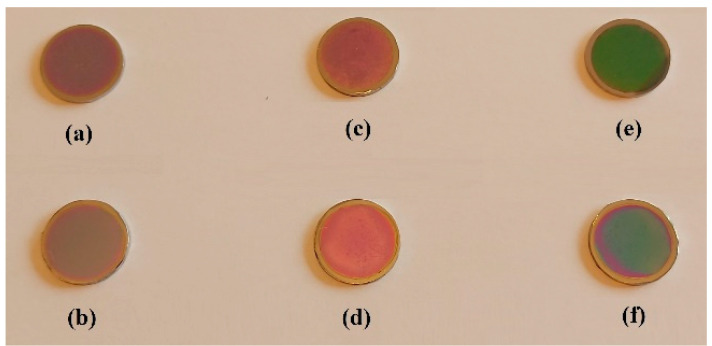
Samples of Co-Cr alloy sintered using DMLS technology and coated with thin films: (**a**,**b**) samples S1 and S2 coated with Ta_2_O_5_; (**c**,**d**) samples S3 and S4 coated with 75% Ta_2_O_5_; 25% ZnO; (**e**,**f**) samples S5 and S6 coated with 50% Ta_2_O_5_; 50% ZnO.

All samples were coated with thin films with a thickness of fifteen microns. Two sintered disks (S1 and S2) were coated with thin film of Ta_2_O_5_. Two other sintered disks (S3 and S4) were coated with composite thin film of 75% Ta_2_O_5_ and 25% ZnO and the last two disks (S5 and S6) were coated with composite thin film of 50% Ta_2_O_5_ and 50% ZnO, as shown in Figure 1.

### 3.2. SEM and EDS Analyses of Co-Cr Samples Fabricated by the DMLS Process and Coated with Ta_2_O_5_ by E-Gun Technology

**Figure 2:**
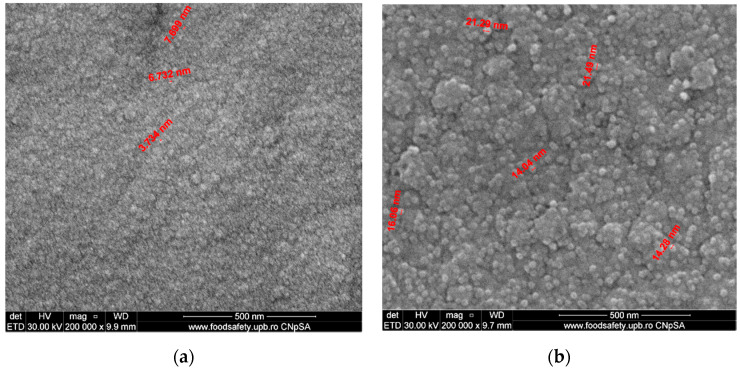
FE-SEM analysis of Co-Cr samples fabricated via DMLS and coated with a thin film of Ta_2_O_5_ produced by an e-gun for: (**a**) sample S1 with post-treatment; (**b**) sample S2 without post-treatment (200,000×).

The microstructure of samples of Co-Cr fabricated by the DMLS process and coated with a thin film of Ta_2_O_5_ using e-gun technology is presented in Figure 2. Figure 2a presents the SEM analysis of sample S1, which was post-treated after the DMLS process and covered with Ta_2_O_5_ film and a very fine and uniform structure with the grains’ dimensions of angstrom order 30 Å, produced by e-gun technology. The roughness obtained was excellent. Sample S2, shown in Figure 2b, without post-treatment after DMLS process, presented a finer structure with the grains’ dimensions between 14 and 21 nm. For sample S1 (Figure 2a), subjected to post-treatment after the DMLS process, the thin film grains were very fine, having the dimensions between 3 and 7 nm. The post-treatment after DMLS process led to the decrease in the surface porosity and permitted better support for e-gun coating with Ta_2_O_5_. The structure in the case of sample S1 shown in Figure 2a was finest compared to the structure of sample S2 shown in Figure 2b.

**Figure 3:**
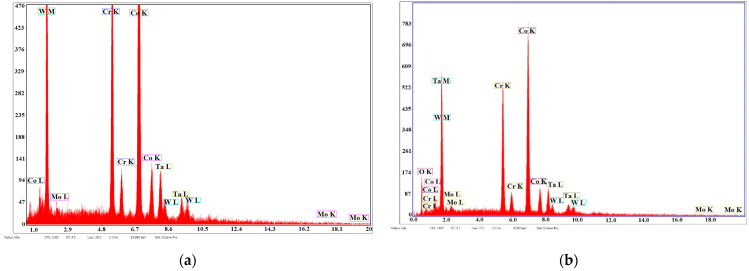
EDS analysis of sintered samples of Co-Cr alloy fabricated via DMLS and coated with a thin film of Ta_2_O_5_ using an e-gun for: (**a**) sample S1 with post-treatment and (**b**) sample S2 without post-treatment.

Figure 3 presents the EDS analyses in the case of samples S1 and S2 and the preponderance of the peaks of Co, Cr, Mo, and W belonging to the substrate and small peaks of Ta and O of the deposited film.

**Figure 4:**
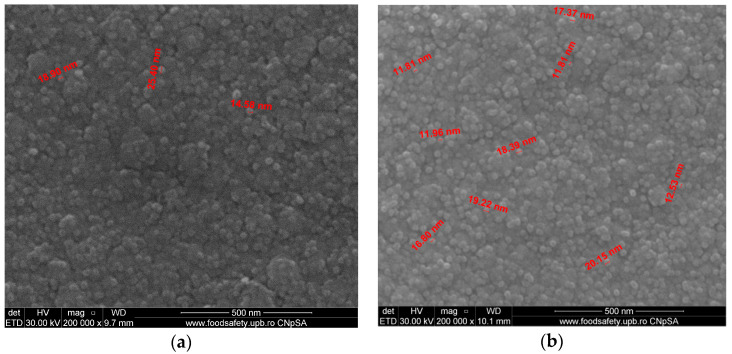
FE-SEM analyses of Co-Cr alloy samples fabricated by DMLS and coated with composite thin film of Ta_2_O_5_ (75%) and ZnO (25%) by e-gun: (**a**) sample S3 without post-treatment, (**b**) sample S4 with post-treatment (200,000×).

The FE-SEM analyses of both sintered samples of Co-Cr alloy fabricated by the DMLS process and coated with a thin composite film of Ta_2_O_5_ (75%) and ZnO (25%) using e-gun technology are presented in Figure 4. In Figure 4a, the grains are uniformly distributed and the dimensions of grains are between 11 and 20 nm in sample S3, which was post-treated in the oven for 30 min after DMLS process. For sample S4 (Figure 4b), the structure was less fine compared to sample S3 and the grain dimensions were between 14 and 25 nm, much larger compared to that attained by applying post-treatment after the DMLS process.

The structure of the composite film was less fine compared to the structure of pure Ta oxide film, shown by FE-SEM analyses (Figure 2 and Figure 4).

**Figure 5:**
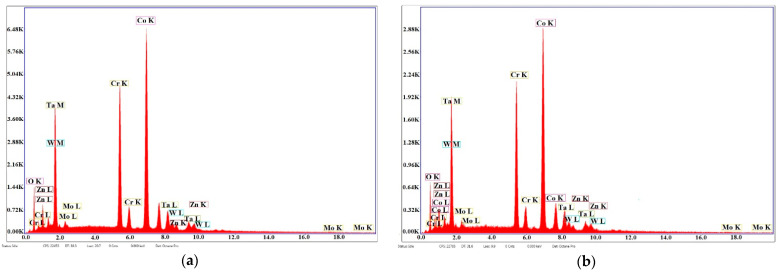
EDS analyses of Co-Cr alloy samples fabricated by DMLS and coated with composite thin film of Ta_2_O_5_ (75%) and ZnO (25%) using an e-gun: (**a**) sample S3 with post-treatment; (**b**) sample S4 without post-treatment.

Figure 5 presents the EDS analyses of samples S3 and S4 and the peaks of Co, Cr, Mo, and W of the superalloy and peaks of Ta, Zn, and O, which show the presence of Ta and Zn oxides on the thin deposited film.

**Figure 6:**
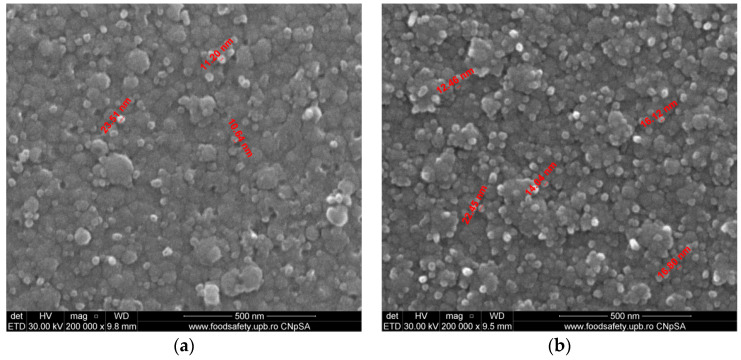
FE-SEM analyses of samples composed of Co-Cr alloy produced by DMLS and coated with a thin film of Ta_2_O_5_ (50%) and ZnO (50%) using an e-gun: (**a**) sample S5 without post-treatment; (**b**) sample S6 with post-treatment (200,000×).

Figure 6 presents the FE-SEM analyses of both sintered samples of Co-Cr alloy fabricated by the DMLS process and coated with thin composite film of Ta_2_O_5_ (50%) and ZnO (50%) using e-gun technology.

Figure 6a presents the composite thin film 50% Ta_2_O_5_ and 50% ZnO of sample S5. The granulometry comprised between 10.64 and 23.51 μm with uniform deposition. Figure 6b shows the deposition layer produced by e-gun technology with 50% Ta_2_O_5_ and 50% ZnO in the case of sample S6. The diameters of the grains were between 12.46 and 22.45 μm. Sample S5, shown in Figure 6a, presented better granulometry, finer compared to sample S6 shown in Figure 6b, because sample S5 has been supplementary post-treated for 30 min in the oven after DMLS process.

**Figure 7:**
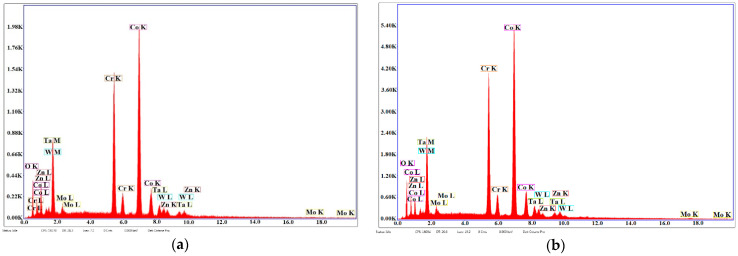
EDS analyses of samples of Co-Cr alloy fabricated by DMLS and coated with a thin film of Ta_2_O_5_ by an e-gun for: (**a**) sample S5 with post-treatment; (**b**) sample S6 without post-treatment.

Figure 7 presents the EDS analyses for samples S5 and S6; the predominant peaks of Co, Cr, Mo, and W in the structure; and an eloquent increase in the peaks of Zn and O and a decrease in Ta peaks that were found in the structure of the thin film.

### 3.3. XRD Analysis of Sintered Samples of Co-Cr Alloy by the DMLS Process and Coated with Thin Films by E-Gun Technology

**Figure 8:**
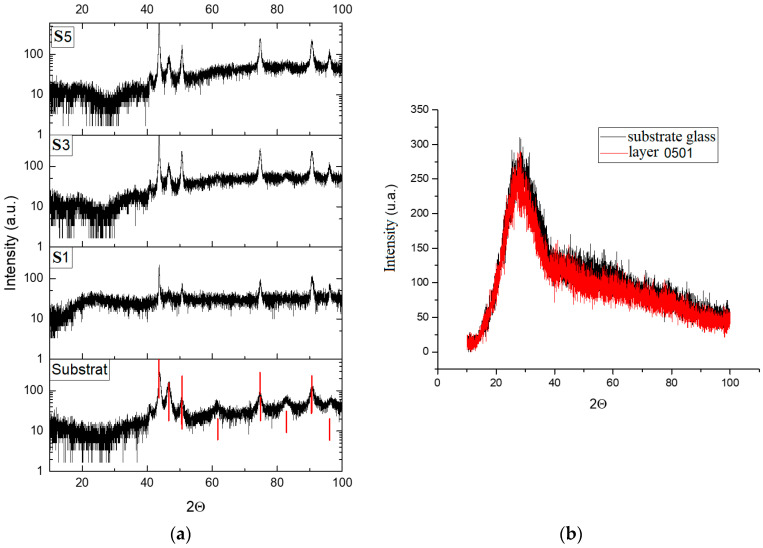
XRD analyses of sintered samples and coated using e-gun technology. (**a**) XRD spectra of thin film deposition on the glass substrate in case of samples S1, S3 and S5; (**b**) XRD spectra of thin film deposition on two glass samples in similar conditions like in the case of samples S1 and S3.

XRD analysis, presented in Figure 8a, was performed on samples S1, S3 and S5, which were subjected to thermal post-treatment after DMLS process in the oven, with the results being reported using one standard sample composed of glass material. From the analysis of the spectra shown in Figure 8a, there are no clear peaks belonging to the layer produced by the e-gun technique. All visible peaks are present for the sample (measurement performed on the back of S1).

The conclusion in this case was that the layers were amorphous. To verify this conclusion, the XRD spectra were recorded for the layers deposited on an amorphous glass substrate. The results shown in Figure 8b confirm the initial conclusion that the thin films were amorphous. All samples presented peaks of Co, Cr, and Ta, while samples S3 and S5 only have presented Zn peaks.

### 3.4. AFM Analyses of the Surface Morphology and Roughness

The surface morphology and roughness were evaluated by AFM analyses of samples S1, S3, and S5. The results are presented in Table 1.

AFM analysis of the surface morphology and roughness of Ta_2_O_5_ deposited by e-gun on sample S1 of Co-Cr alloy fabricated by DMLS, with post-treatment, presented very low roughness of angstrom in the order Ra = 19.7 Å and Rrms = 26.6 Å, in concordance with the results shown in Figure 2.

For sample S3, the analysis conducted on the same substrate, for the post-treated sample, coated with a composite thin film of Ta_2_O_5_ (75%) and ZnO (25%) by e-gun showed an increase in roughness to Ra = 6.11 nm and Rrms = 8.24 nm, in concordance with the results shown in Figure 4.

In the case of sample S5 fabricated by DMLS, with post-treatment, and coated with composite thin film of Ta_2_O_5_ (50%) and ZnO (50%) by e-gun, the AFM analysis revealed higher roughness of Ra = 14.8 nm and Rrms = 20.70 nm.

In conclusion, the roughness increases with the amount of ZnO in the coatings, being higher for samples S3 and S5 compared to the sintered sample S1, which was coated only with Ta_2_O_5_. Both SEM and AFM surface analyses showed an increase in roughness of the c samples coated with ZnO by an e-gun. The increase in roughness was further enhanced by the thermal post-treatment of the samples, as shown in the SEM images presented in Figure 6.

### 3.5. Hardness and Modulus of Elasticity Determined Using the Nanoindentation Method

**Figure 9:**
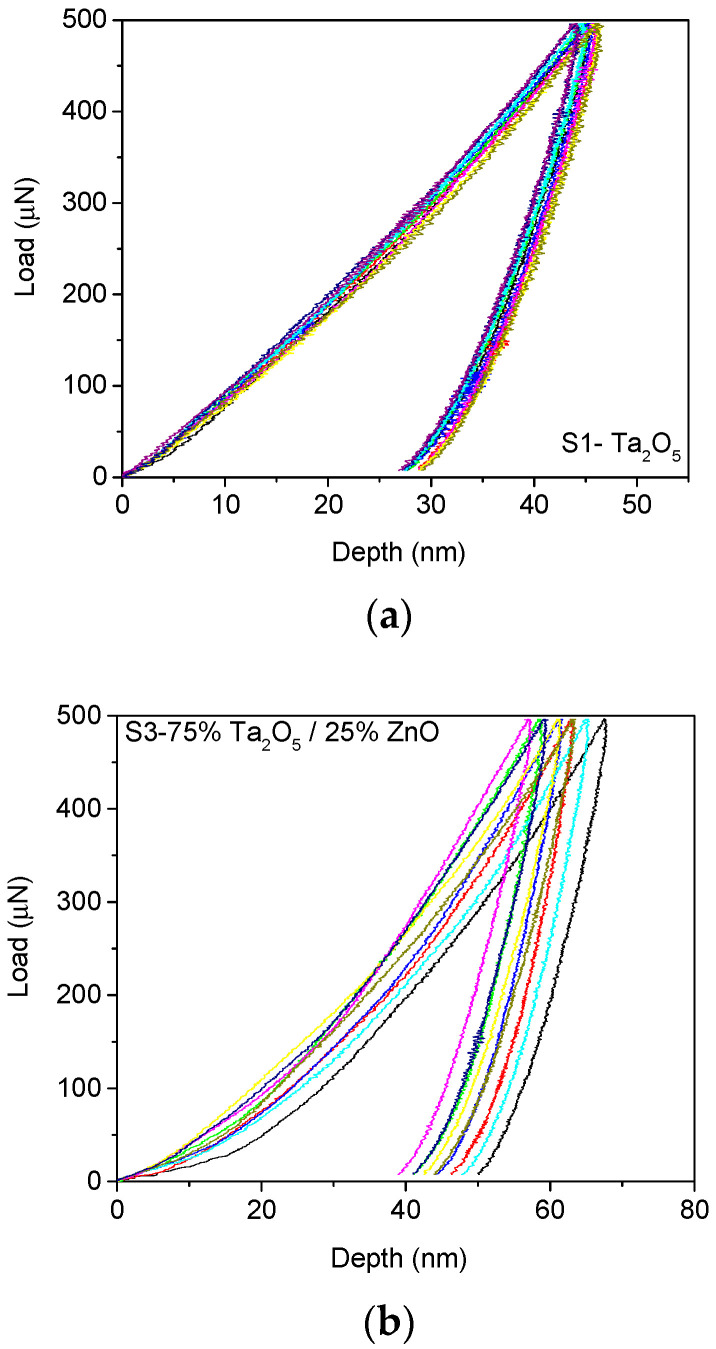

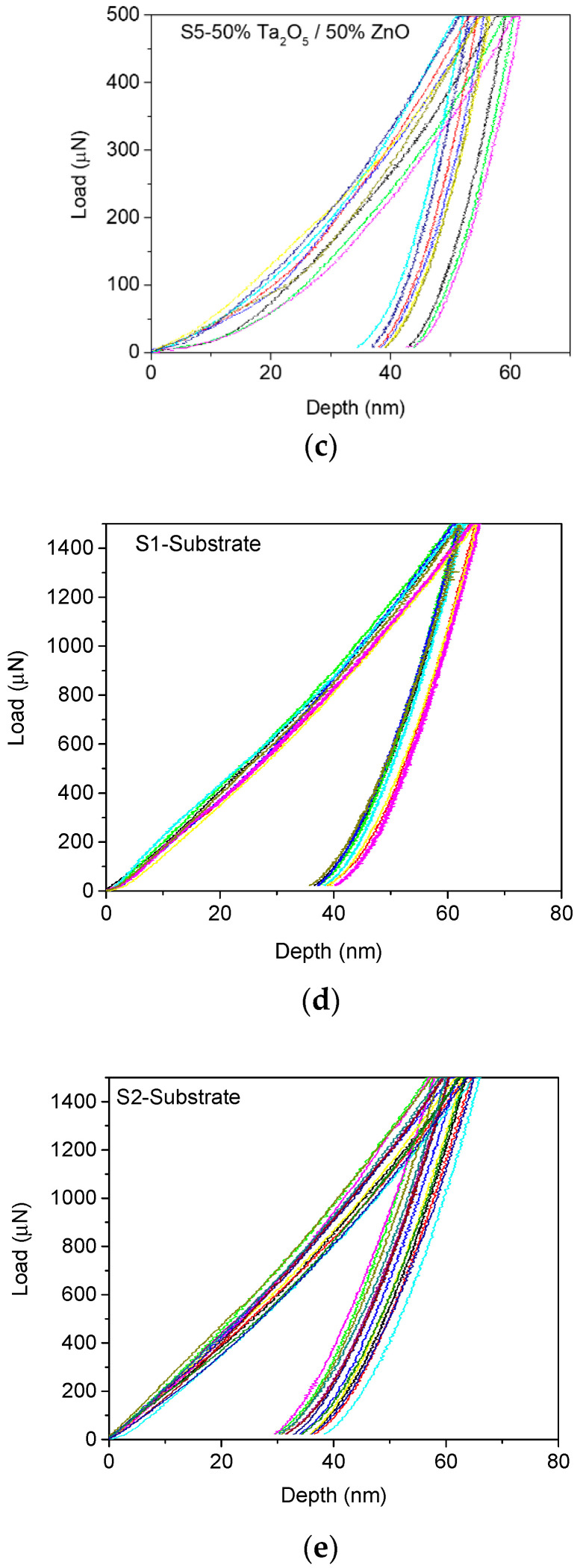
The nanoindentation curves of the coated samples (**a**) S1, (**b**) S3, and (**c**) S5. The nanoindentation curves of the Co-Cr base layer for samples (**d**) S1 and (**e**) S2.

The nanoindentation curves used to calculate the hardness values and the reduced modulus for samples S1, S3, and S5 are shown in Figure 9. The maximum loading force was chosen so that the mean contact depth surpassed the minimum 40 nm range, required by the geometry and the calibration of the tip.

The results concerning the hardness and reduced modulus of the thin film deposited by an e-gun for samples S1, S3, and S5 are presented in Table 2. In Table 2, the highest hardness value was obtained with sample S1, which was coated with Ta_2_O_5_. The addition of ZnO significantly decreased the hardness to 2.95 GPa, whereas a further addition of ZnO led to a moderate increase to 3.37 GPa.

The nanoindentation curves for Co-Cr samples S1 and S2 coated by Ta_2_O_5_ are presented in Figure 9e. In these cases, the hardness and modulus values for the Co-Cr base layer were significantly reduced, as presented in Table 2. As shown in this table, the hardness values were significantly higher, by a factor of two, compared to the values of the hardness that were reached in the case of oxide coatings. Table 2 shows that the average values of hardness in the case of Co-Cr alloy substrate varied between 9.594 and 10.549 GPa, while the average values of the modulus varied between 189.637 and 206.789 GPa.

## 4. Conclusions

Ta and Zn coatings are important for improving the surface hardness and surface roughness of implants to ensure the in vitro bioactivity and biocompatibility of these parts. In this article, we produced Ta_2_O_5_ and ZnO coatings using e-gun technology with Co-Cr samples fabricated with the DMLS process with the aim of improving these important characteristics. Disk samples were fabricated of Co-Cr material using the DMLS method and one post-treatment process applied in a furnace for 30 min at 900 °C to determine if this process influences on the following e-gun coating. From the SEM and EDS analyses, we found that the post-treatment applied after the DMLS process led to the decrease in the surface porosity and provided better support for the e-gun coating of the samples with Ta_2_O_5_. Through SEM analyses, we determined the thin films depositions of Co-Cr samples fabricated by DMLS and the fine granulometry of Ta_2_O_5_ deposition at the angstrom order. The deposited composite thin films (Ta_2_O_5_ (50%) and ZnO (50%)) presented uniform grains, having the diameter of the nanometer order. XRD analysis was used to determine the chemical composition of the sintered samples and showed that the thin films were amorphous. AFM tests showed that the roughness of the Ta_2_O_5_ and ZnO films deposited on the sintered Co-Cr samples was Ra = 19.7 Å, confirming that a very fine structure of the deposited Ta_2_O_5_ layer was produced using this method. The nanoindentation tests were used to determine the hardness values and reduced modulus of the sintered samples and Ta_2_O_5_ coating, with the hardness being in the order of 4.7 GPa and the elasticity modulus in the order of 118.533 GPa. All three types of thin films that were studied in this article can be used to improve the characteristics of implants fabricated of Co-Cr implants using DMLS to determine their benefit on cell–biomaterial interactions.

## Figures and Tables

**Table 1:** Surface morphology and roughness realized by AFM analyses.

Sample	Scanned Area (µm^2^)	2D Image View of the Layer	3D Image View of the Layer	Roughness Parameters	
				Ra (nm)	R_rms_ (nm)
S1_layer	5	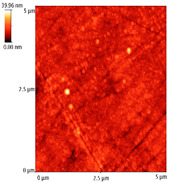	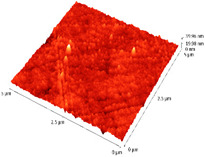	1.97	2.66
	10	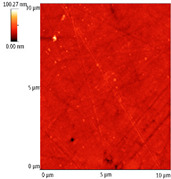	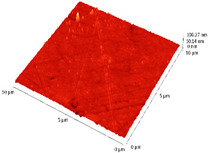	2.19	3.23
S3_layer	5	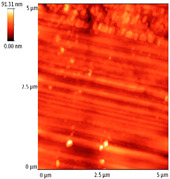	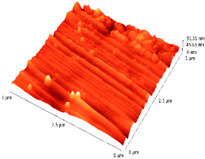	6.11	8.24
	10	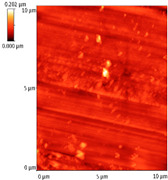	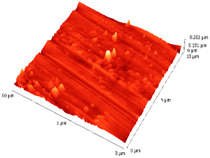	8.90	12.80
S5_layer	5	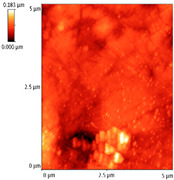	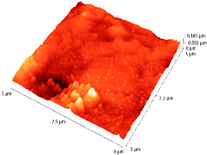	14.80	20.70
	10	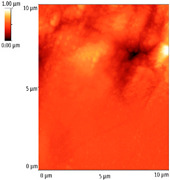	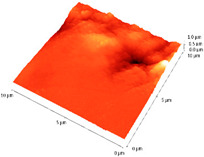	54.00	88.50

**Table 2:** Hardness values and modulus of elasticity calculated for thin film and for Co-Cr base layer using nanoindentation curves.

Sample Zone Layer Force	Average Contact Depth (nm)	SD of Contact Depth (nm)	Average Hardness (GPa)	SD of Hardness (GPa)	Average Reduced Modulus (GPa)	SD of Reduced Modulus (GPa)
S1_substrate_0v5mN	37.365	0.849	4.703	0.127	118.533	4.079
S3_substrate_0v5mN	54.306	3.397	2.95	0.242	98.846	9.5
S5_substrate_0v5mN	49.231	3.229	3.372	0.283	116.376	6.968
S1_substrate_1v5mN	51.211	1.506	9.594	0.363	206.789	6.977
S2_substrate_1v5mN	47.731	2.892	10.549	0.822	189.637	5.833

## Data Availability

No new data were created or analyzed in this study. Data sharing is not applicable to this article.
